# On Evans' Amalgam for Filling Teeth

**Published:** 1850-01

**Authors:** J. Robinson

**Affiliations:** European Correspondent.


### ARTICLE VII.

*On Evans' Amalgam for Filling Teeth.* *By* J. ROBINSON, D. D. S.—European Correspondent.

No question has occupied so much of the attention of the scientific and learned professors of the dental art, in the present day, as the discovery of a substitute for gold foil, the only recognised substance employed by intelligent practitioners, as it is the only effectual mode of plugging the cavities of carious teeth. The difficulties attending the use of gold are so obvious, that it is not necessary that much patient investigation should have been bestowed, and many interesting analytical experiments with regard to the chemical combination and action of various mineral and metallic substances, should have been tried, to discover a substitute that would embrace all the necessary requirements. We regret to say, that up to the present time, the results have been extremely questionable and unsatisfactory.

It is not our intention on the present occasion to point out the various methods resorted to by the leading practitioners in this country, and abroad, for introducing the foil into a cavity, and securing it permanently. Whatever may be the peculiarity of the *modus operandi*, all are unanimous upon one point, that to plug teeth with gold, a large amount of physical and mechanical exertion is required, while to add to the difficulty, it is undeniable that unless certain important preliminary conditions are observed in preparing, and subsequently in plugging, the cavity, the ultimate success and durability of the operation are extremely questionable. Nor is this all. Either from a want of proper mechanical skill, or ignorance of the deleterious effects of amalgams, or an unwillingness to undergo the amount of physical labor and exertion which gold plugging requires, or from still more discreditable and unworthy motives, many unsuspecting patients have become the victims of the grossest impositions, and the prey of malignant disease from the indiscriminate and improper use of compounds, containing large quantities of mercury, silver, tin, bismuth, &c., in various combinations and proportions. The use of these metallic compounds, is, unfortunately, much more general than could be imagined by persons unacquainted with the subject, and it is to be regretted, that the malpractices of these dental empirics who resort to them, have, in many instances, been the exciting cause of the most painful and dangerous diseases, terminating, not unfrequently when the constitution has been otherwise impaired, in death.

The extent to which the practice of introducing mineral compounds and amalgams prevails, not only in this country, but on the Continent, and among our American brethren, may be estimated by the fact, that the Society of American Dentists, much to their honor, took the initiative in repressing the practice, and expressed their determination to exclude from their society, any member who employed them. This decision of the council excited considerable opposition, and the question was even raised, whether the society itself, had the power to exclude them. But even those who advocated the use of the metallic compound *in certain cases*, admitted that they were ready to forego its use, if a substitute could be found.

Notwithstanding this expression of opinion by the Society of American Dentists, the question of the use of amalgams remains still unsettled. The most eminent dentists in the United States have held, and we believe at present hold, opinions more or less opposed to each other, as to whether amalgams of every description can or cannot with propriety be discarded from the practice of a dentist. The scientific inquirer would naturally prefer leaving the knotty question still open to further research and investigation, avoiding all tampering with those compounds, whose deleterious action is apparent, and using due caution in the application of these, whose negative or innoxious qualities had been ascertained.

Such was apparently the state of things, when at the commencement of the present year, the gentleman whose name is prefixed to this notice, and who is well known for his professional and scientific attainments, announced the discovery of a compound exempt from many of the objections that had been raised to former ones. Mr. Evans' compound, which, with the liberality of all true laborers in the field of science and discovery, he, after a few months' trial, publicly communicated to the profession, was not intended by him as a substitute for gold, although it was generally supposed that such was the case. It was intended to be used solely in those peculiar cases which might seem to indicate the expediency of resorting to a soft filling, and to take the place of those pernicious compounds previously in use. He was under the impression that he had obtained that great desideratum, the discovery of a metallic compound that would not undergo any chemical change in the mouth, and would neither discolor nor disorganize the osseous structure of the tooth. If, indeed, that had been accomplished—if the chemical equivalents were so accurately balanced, that no uncombined mercury existed, and if the attraction between it and the tin and the cadmium, had been such that no oxyd of mercury would be found by the salts of the mouth, Mr. Evans would have been entitled to all the honors of a most important discovery, and the thanks of his professional brethren. Time, however, the great touchstone of novelties and innovations, has proved that this most desirable result remains yet to be accomplished.

No sooner, however, did the discoverer announce to the profession the apparent merits of the new compound, and before the test of experience had been applied to it, than several of those sapient gentlemen to whom nothing is new, came forward, open mouthed, to assure the public that they had previously used a similar compound, but did not think it worth while to communicate it to the profession. When Columbus made the egg stand upon end, all those who had previously failed, exclaimed, that "nothing was more easy." Mr. Evans may console himself, when he is assured by those who are ungenerous and dishonorable enough to attempt to deprive him of the merit to which he is fairly entitled, "that the invention is not his," by the reflection, that Columbus was treated just as scurrily. Others have not been wanting to decry the value of the invention, and have been unsparing in their strictures upon the compound itself and the motives of the discoverer.

While we cannot but express our regret that so much of professional jealousy should have been exhibited in the discussion of a question of so great importance to the interest of dental surgery, and which should be cultivated and carried out in a more liberal and comprehensive spirit, we may state, that it is not our intention to become either the advocates or the apologists of Mr. Evans' or any other amalgam yet discovered. As is well known, we have little, or no faith, in any article, of which mercury forms a component part. We are too well acquainted with the injurious results of the employment of amalgams by many of the professors of the dental art in England, to become their eulogist, or their sponsors. Our duty in the present instance, is the simple one of noticing Mr. Evans' new discovery, and so far as our experience has gone, recording our opinion of its merit.

With this object, we determined to test its efficacy in our practice at a public hospital, before pronouncing any decided approval, or condemnation of the compound. We felt ourselves the more imperatively called upon to take this step, because certain dental wiseacres, to whom we may allude further on a future occasion, had thought proper, after a few times trial, to pronounce a sweeping and indiscriminating eulogium upon the new discovery. We may premise, that we never for one moment believed, or expected, that the compound would be a substitute for the more trusty and efficient filling with gold, and such was the purport of any communications or personal conversations we had with the discoverer. We gave the invention a fair, and we may add, an extensive trial in every case of caries coming within our range at a public institution. We have found, that as a filling, it answers chemically all that the inventor claimed for it at the commencement, but like all compounds containing mercury, we found, that contraction, to a certain extent, subsequently took place, followed in some instances by a loosening of the plug. In no single instance did we discover any blackening of the osseous structure of the tooth, after even six months introduction. In some cases in which the filling had become loose, or where the tooth had been stopped laterally, we observed that a yellow softened appearance of the osseous structure existed, and a similar appearance presented itself in teeth, which, at a subsequent period, it had been found necessary to extract.

Such is the general result of our experiments with Mr. Evans' new compound which we feel it our duty to make public, without, in the slightest degree, wishing to disparage the efforts of the eminent scientific gentleman, who has labored so industriously for the common good of the profession. Although his expectations have not been realized to their full extent, Mr. Evans has done good service in introducing what appears to be at least an innoccuous compound as a substitute for the pernicious and dangerous amalgams hitherto used. We are gratified to find, that after some months of close observation and investigation, Mr. Evans has had the courage and the manliness to come forward and avow his own dissatisfaction in the following words:

"It appears to have been inferred from my remarks in several English medical journals, that I am an advocate of the practice of filling teeth with amalgams. This is an honor to which I have not the slightest claim whatever, and I must therefore leave it entirely to the enjoyment of those who are justly entitled to it. The fact is well known to my friends on this side of the Atlantic, as well as in America, that I have always been strongly opposed to the use of any other material for filling teeth than gold, and in my own practice I have never made use of any other. My predominant opinion has always been decidedly in favor of gold, notwithstanding I have, in common with others, experimented with a view, on my part, of testing the merits of substances designed to be used in a plastic state. From my experiments with the preparation alluded to, I discovered that it had qualities which the other amalgams did not possess. It preserves its color better than the other mercurial preparations I am acquainted with. It also has the advantage of becoming a tough ductile substance, susceptible of being cut or burnished like a piece of tin. In addition, the cadmium seems to completely absorb the mercury in the process of crystalization. From these circumstances, it was thought to possess important advantages over any of the substances hitherto employed.

There is, in my opinion, a very great objection to all the combined metals, arising from the fact that they are all promoters of galvanic action. This objection is a very serious one in a substance employed for filling teeth. Upon removing some of this filling, which appeared perfectly tight, and which was unchanged in its color upon the exterior surface, I have found that beneath the metal a deep yellow hue had made its appearance, penetrating for some distance into the bone of the tooth. This phenomenon does not present itself until after the lapse of considerable time, and appears much more striking in some cases than others. Whether this effect is to be ascribed to galvanic agency yet remains to be determined. Mr. Faraday observes, (see Turner's Chemistry, sixth American edition, page 399,) that an alloy of steel with one-hundredth part of its weight of platina, dissolves with effervescence in diluted sulphuric acid, so weak as scarcely to act on common steel; a fact which he accounts for by ascribing it to the steel being rendered positive by the presence of the platina.

I have watched the effects produced by the application of this preparation, and the result of my observation has been such as I have stated above. I am now engaged in investigating the subject of the different amalgams that have been in use, in reference to their influence upon the general health; and I hope soon to be able to give the opinion of some of the highest medical authorities in Europe, upon a question which has elicited so much discussion, and which, in reality, is one of the greatest importance.

In giving publicity to this preparation, the object was not simply to make known the result of my experiments, but also to invite the attention of the profession to a subject so peculiarly important as that of discovering a material to be used *in those cases in which the circumstances might seem to indicate the expediency of employing a soft filling*, and to take the place of the compositions deemed objectionable, already in use. Experience, the great test in such matters, has furnished a result not so favorable as was anticipated by many members of the profession, who had expressed their decided approbation of this preparation, and who seemed perfectly confident that it was destined to fill a great desideratum which had so long been felt.

Whatever may be the hopes of others in regard to this preparation, *I* have *never* considered it as a substitute for gold—I have regarded it as a mere expedient, to be resorted to only in *peculiar* cases.

In regard to its influence upon the general health, in the opinion of those whose authority is entitled to weight, it is not injurious in this respect. *I cannot, however, refrain from stating it as my deliberate opinion, that all operations in which amalgams are employed are merely temporary in their nature, and that any tooth that can be filled in a* PROPER *manner with* GOLD, *can be effectually and permanently saved only by this means.* This is the opinion which I have always entertained, and I adhere to it at the present moment with undiminished confidence." Yours, respectfully,

THOS. W. EVANS.

To Messrs. Jones, White & Co.

The discovery of Mr. Evans, and the frankness with which he subsequently acknowledges its defects is another practical illustration of the utility of some guiding principle in the conducting of inquiry, and of the assistance which even the failures of those who are animated by the true spirit of investigation may afford in giving a more profitable direction to the labors of others. For this, alone, Mr. Evans is entitled to the thanks and gratitude of his professional brethren. The attention of thinking and intelligent minds is directed thereby to the immediate causes of success or failure—new lines of thought are struck out, new relations and combinations are suggested, the interests of dental surgery are promoted, in this, as in every other instance, and the inculcation of every new fact and experiment must inevitably result in the advancement of our general knowledge and acquirements.

---

